# Dynamic analysis of Tapping Panel Dryness in *Hevea brasiliensis* reveals new insights on this physiological syndrome affecting latex production

**DOI:** 10.1016/j.heliyon.2022.e10920

**Published:** 2022-10-03

**Authors:** Eva Herlinawati, Pascal Montoro, Sigit Ismawanto, Afdholiatus Syafaah, Martini Aji, Michel Giner, Albert Flori, Eric Gohet, Fetrina Oktavia

**Affiliations:** a Sembawa Research Centre, Indonesian Rubber Research Institute, Palembang, Indonesia; b CIRAD, UMR AGAP Institut, F-34398 Montpellier, France; c UMR AGAP Institut, Univ Montpellier, CIRAD, INRAE, Institut Agro, F-34398 Montpellier, France; d CIRAD, UPR AIDA, F-34398 Montpellier, France; e CIRAD, UMR ABSys, F-34398 Montpellier, France; f Sungei Putih Research Centre, Indonesian Rubber Research Institute, Deli Serdang, Sumatera Utara 20585, Indonesia

**Keywords:** Dry cut, Marker, Phenotyping, Rubber, Sucrose, Yield

## Abstract

Tapping Panel Dryness (TPD) is a physiological disorder affecting natural rubber production in *Hevea brasiliensis*. TPD is associated with clonal susceptibility and overexploitation of rubber trees. Most studies are based on a binary point view of the absence or presence of TPD. This study sets out to characterize the dynamic of the TPD onset through the monthly monitoring of the dry cut length. This reveals the presence of dry spots on the tapped panel of any trees. The frequency of these dry spots increases dramatically in trees developing high level of TPD. Brown bast is an irreversible form of TPD. Brown bast is correlated to a high level of dry cut length. Application of an intensive harvesting system induces early TPD occurrence, which facilitates the study of TPD. Among latex diagnosis parameters, only sucrose content is significantly associated with TPD. Other parameters are more prone to environmental effects and are not reliable as physiological markers. These findings explain the contradictory conclusions of some papers. This study suggests to use intensive harvesting system and monitor the dry cut length for genetic analysis of TPD.

## 1 Introduction

Natural rubber (NR) is a renewable product, which plays an important role in the economy of producing countries. *Hevea brasiliensis* is the main source of NR. This *cis*-1,4-polysioprene is synthesized in the rubber particles of laticifers. These latex cells are articulated and anastomosed to create a cellular network enabling the production of a large volume of latex after cutting, also called tapping, the soft bark of rubber trees without damaging the vascular cambium. Complete latex regeneration required 48–72 h depending on clone latex metabolism. For some rubber clones, the application of an ethylene releaser, the ethephon or 2-chloroethylphosphonic acid, on the tapping panel is necessary to increase both the latex flow after tapping and latex regeneration between two tappings. Harvesting systems, frequency of tapping and ethephon application, are dependent on the latex metabolism (Jacob et al., 1989b). Many parameters were used to describe the hysiological status of laticifers. In rubber estate plantations, harvesting system recommendations to optimize the latex production are basedon the latex diagnosis (LD), which consists in measuring four main parameters: sucrose, inorganic phosphorus (Pi) and reduced thiol (RSH) contents, as well as the total solid content (TSC) in latex (Jacob et al., 1989a). Sucrose is the source of energy and carbon for the rubber biosynthesis. Pi content reflects the energy metabolism including the turn-over of ATP (Jacob et al., 1989b). RSH content is associated with the detoxification capacity of laticifers. Lastly, TSC allows estimating the dry rubber content. This latex diagnosis leads to define a clonal typology used to classify rubber clones and to predict their most suitable harvesting system (Jacob et al., 1995; Lacote et al., 2010).

NR production is dramatically affected by Tapping Panel Dryness (TPD) (Commère et al., 1989). TPD is a physiological disorder resulting from excessive recurrent tapping and overstimulation by ethephon (Jacob et al., 1994; Obouayeba et al., 2012; Yusof et al., 1995), rootstock-scion incompatibility (Sobhana et al., 2005), or soil compaction (Nandris et al., 2004), as well as seasonal effect (Eschbach et al., 1984; Okoma et al., 2011; Van De Sype, 1985). In a context of climate change, abiotic stress susceptible rubber clones are supposed to be prone to higher TPD occurrence. This physiological syndrome is characterized by two kind of symptoms, ROS-TPD and BB-TPD described by Putranto and collaborators (Putranto et al., 2015). The first symptom is associated with an overproduction of reactive oxygen species (ROS). This oxidative stress in latex cells promotes the peroxidation of the membrane of vacuo-lysosomal particles called lutoids, which release notably Hevein, an agglutin protein (Chrestin et al., 1984). This process leads to the agglutination of rubber particles that impedes latex flow after tapping (for review (Zhang et al., 2017)). ROS-TPD-affected trees can recover a normal latex flow after a resting period (Jacob et al., 1994). The second symptom associates both an irreversible cessation of latex flow and brown bast (BB). BB-TPD deals with a deformation of bark due to thylosoid formation, lignified gum, and abnormal division of parenchyma cells (de Faÿ and Jacob, 1989). Some authors described other physiological diseases called bark or trunk phloem necrosis (Lertpanyasampatha et al., 2014; Nicole et al., 1991). According to de Faÿ and collaborators, these physiological diseases are identical or a variant of TPD (de Faÿ, 2011). TPD-affected trees can be sometimes cured by bark scraping and application of chemicals in order to promote bark renewal. However, this process is costly and not widely used.

Breeding for TPD-tolerant rubber clones is impeded by a long phenotyping period and a weak knowledge of molecular and genetic bases of TPD. Little is known about the phenotypic variability for TPD tolerance. A sufficient number of TPD-affected trees can be only observed in a population after 10–15 years of cultivation consisting of an immature period before production of 4–7 years according the clone growth, and several years of mature period of production. Although TPD may be governed by heritable genes (Narayanan and Mydin, 2014), environmental influence makes difficult to study the heritability of this trait (Omokhafe and Aniamaka, 2000). In order to unravel molecular mechanism underlying TPD, many studies were conducted these last years at the transcriptomic and proteomic levels both in latex and bark tissues (Li et al., 2016; Liu et al., 2015, 2021; Montoro et al., 2018; Nazri, 2020; Yang et al., 2018). TPD occurrence is associated with the expression of hundreds of genes notably involved in ROS metabolism, programmed cell death, rubber biosynthesis and the response to ethylene, jasmonate and wounding. Nevertheless, this literature remains descriptive and have not clearly demonstrated the involvement of these candidate genes. To date, the literature on physiological mechanism of latex agglutination is relevant and led to some functional models (Berthelot et al., 2014, 2016; Wititsuwannakul et al., 2008a, 2008b, 2008c). Besides the role of redox metabolism, relationship between cyanogenesis and latex stability was determined at the onset of irreversible TPD (de Faÿ et al., 2010; Moraes et al., 2014). Some authors observed an increase in TPD when latex thiol, inorganic phosphorus and pH value decreased (Guo et al., 2016). Conversely, some papers suggested that sucrose (Gohet et al., 2019), or Pi and peroxidase activity might be a better stress indicator associated with TPD (Tistama et al., 2019).

Most of these studies rely on binary comparison between trees affected or not by TPD. A first dynamic analysis of TPD occurrence was attempted on a production potential trial with three rubber clones (Putranto et al., 2015). Putranto and collaborators showed that the high latex metabolic clone PB260 was susceptible to TPD whatever the used harvesting system, and the occurrence of TPD was accelerated under high tapping frequency with ethephon stimulation. By contrast, the low latex metabolism clone SP 217 was tolerant to TPD. This latter was affected by TPD only after two years of a daily tapping with ethephon stimulation. These results raise several questions. First, can the application of the d1 ET 12/y harvesting system be a way to screen *Hevea* populations to determine their level of susceptibility to TPD? Second, are high latex metabolism clones such as PB 260 more susceptible to TPD than low-medium latex metabolism clones? Finally, can a LD be an indicator of TPD susceptibility? To answer to these questions, a new polyclonal trial (PT2) was built with five rubber clones including high latex metabolism clones (PB 260, RRI 112 and IRR 118) and 2 intermediate latex metabolism clones (BPM 24 and RRIC 100). In this paper, data of this trial (PT1) were re-analysed as well as data from PT2 conducted with a high tapping frequency and ethephon stimulation in order to validate the ability of this treatment to early induce the occurrence of TPD. The dry cut length (DCL), latex yield and LD were monthly monitored and analysed to better understand the onset of the TPD. This study revealed several patterns of TPD occurrence, challenged the perception of this physiological syndrome and validate the high tapping frequency and stimulation d1 ET 12/y as suitable method for further genetic analysis of TPD. Finally, the intrinsic sucrose content value of clones was negatively associated with TPD.

## 2 Materials and methods

### 2.1 Polyclonal trial PT1

The polyclonal trial PT1 was established at the Sembawa Research Centre of the Indonesian Rubber Research Institute (2°57′42″S 104°30′30″E). This trial planted in 2004 was opened in December 2009. PT1 consists of 3 rubber clones (PB 260, RRIM 600 and SP 217). The tapping panel was tapped as a half spiral tapping (S/2). For each clone, three replicates of one tree each were observed for each of the nine combinations (3 tapping × 3 ethephon frequency levels). Trees were tapped every day (d1), 2 days (d2) and 4 days (d4). Ethephon was applied at 2.5% on the groove of the tapping panel of the bark at various frequencies (0 (0/y), 12 (12/y) and 24 (24/y) times a year).

A partial dataset with LD data from March to May 2012 was published by Putranto and collaborators (Putranto et al., 2015). Supplementary Table 1 contains a full dataset with monthly data yield, LD and DCL data recorded for 3 years.

### 2.2 Polyclonal trial PT2

The polyclonal trial PT2 was established at the Sembawa Research Centre of the Indonesian Rubber Research Institute (GPS coordinates: 2°57′25″S 104°30′30″E). This trial planted in 2012 was opened in December 2016. PT2 consists of 5 rubber clones (BPM 24, IRR 112, IRR 118, PB 260, RRIC 100) including two new clones with high latex metabolism (IRR 112, IRR 118), in order to test the hypothesis of TPD susceptibility of these types of clones as clone PB 260. The tapping panel was tapped as a half spiral tapping (S/2). For each clone, fifteen trees were subjected to the tapping frequency d1 (6 days a week) and ethephon 2.5% applied on the groove of the tapping panel 12 times a year. Supplementary Table 2 contains a full dataset with data for latex yield, LD and DCL data monthly recorded for 2 years.

### 2.3 Determination of the DCL, latex yield, LD parameters and brown bast

Tapping panel dryness was assessed by visual assessment of the percentage of DCL instantly after tapping before latex flows on the groove. DCL was observed once a month by the same observer in order to avoid variability between observers. The latex yield was measured by weighing the cup lump from each tree per tapping. After tapping, latex was left uncollected and allowed to coagulate in the collecting cup overnight to form cup lump. Cup lump was collected and weighed the next day before subsequent tapping. The rubber yield was calculated based on the dry rubber content (DRC) measurement of the cup lump that was carried out periodically. Latex diagnosis parameters (sucrose, Pi, RSH and TSC) were monthly measured 48 h after the last tapping and 3 weeks after the last ethephon stimulation in order to avoid any variation due to these harvesting stresses. Fresh latex (0.5 ml) was supplemented with 4.5 ml of 2.5% trichloroacetic acid (TCA) to induce protein precipitation, and then the latex serum was filtered with filter paper. This clear latex serum was then used for the determination of sucrose, inorganic phosphorous and reduced thiols contents using the anthrone method (Dische, 1962), the molybdate ammonium method (Taussky and Shorr, 1953), and the acid dithiobisnitrobenzoate (DTNB) method (Mc Mullen, 1960), respectively. TSC was measured by drying five grams of latex sample at 100 °C to a constant weight. TSC was calculated based on the ratio between dry weight and fresh weight of latex. Finally, the number of brown bast spots was counted after scraping bark below the tapped bark up to the graft.

### 2.4 Database and data analyses

All data were recorded on the Microsoft ACCESS database management system. Specific tables were created for each series of variables (T_BROWN_BAST, T_DCL, T_HARVESTING_SYSTEM, T_LATEX_ANALYSIS, T_LATEX_YIELD, T_PLANT_MATERIAL, T_TRIAL, T_TRUNK). Relationships were established in order to combine data from different tables. Each relationship consists of fields of two tables with corresponding data. Queries were designed to extract dataset presented in Supplementary Tables 1 and 2. This database enables tracing and insuring the integrity of this work. K-means clustering, multiple correspondence analysis, ANOVA, principal component analysis (PCA) and linear regression were performed with XLSTAT 2020 (XLSTAT statistical and data analysis solution. Addinsoft, New York, USA. https://www.xlstat.com).

## 3 Results

The effect of tapping and ethephon stimulation frequencies was monitored tree per tree thirteen times during the three years of the PT1 trial for DCL, and once at the end of the trial for the brown bast (Supplementary Table 3). Trees were split into 3 classes using their monthly DCL values for the 3-year period by the k-means clustering method: high DCL (class 1), medium DCL (class 2), low DCL (class 3). None of the 74 studied trees have showed 0% DCL for all the observations during this 3-year trial.

Class 3 consist of trees with a mean DCL lower than 20%. For trees with the lowest average of DCL at 1% (PT1G33, PT1G93, PT1F81, PT1F62 and PT1F92), a partial low DCL was recorded for a period of time suggesting a localized small dry spot. Another tree of the class 3 (PT1F12) with a mean DCL value at 7% had a transient high DCL peaking at 50% revealing larger dry spot.

For trees of class 2, DCL mean values range between 30% and 70%. For these trees, a transient high DCL can be observed more than thrice ranging from 50% to 100%.

For trees of class 1, the mean of DCL is higher than 50%, except for tree PT1F43, which has contrasting DCL data (0–10% or 78–96%). The bark of ten trees was scraped at the end of the trial. Of the seven trees of class 1, three did not show any brown bast spot showing that BB does not occur systematically in high DCL trees.

Supplementary Table 3 shows a contrasting pattern of DCL between clones and the effect of harvesting system. In order to characterize the effect of each factor in this trial PT1, mean values of DCL, latex yield, and latex diagnosis parameters for the 15 trees per clone during all the three-year period were computed and submitted to three-way ANOVA including factors clone, tapping frequency, ethephon stimulation, and their interactions (Supplementary Table 4).

The ANOVA table reveals that DCL, latex yield and some LD parameters are influenced by several factors (Table 1). DCL, latex yield, Pi and TSC depend on 5 to 3 significant factors while sucrose and RSH depend only on factor clone. As the design is balanced and the factors have the same degrees of freedom (3 clones, 3 tapping frequency, 3 stimulation frequency), ANOVA F values can be used to compare effect intensity between the different factors. The factor clone is the most important for DCL, Pi, sucrose and TSC with F equals to 99.6, 66.1, 38.7, and 37.4 respectively, and to a lesser extend for RSH (F = 5.1). For yield, the highest F is observed for the tapping factor (F = 138.6).

**Table 1:** ANOVA interaction effect of factors (clone, tapping and ethephon frequencies) on the variables (DCL, latex yield, and latex diagnosis parameters) for the trial PT1.

	DCL		Yield		TSC		Suc		Pi		RSH	
Source	F	Pr > F	F	Pr > F	F	Pr > F	F	Pr > F	F	Pr > F	F	Pr > F
Clone	**99.6**	1.88245E−34	**5.0**	0.007	**37.4**	3.42227E−15	**38.7**	0.000	**66.1**	0.000	**5.1**	0.006
Tapping	**40.3**	2.40964E−16	**138.6**	0.000	**10.6**	3.74404E−05	1.1	0.337	**4.7**	0.010	0.1	0.908
Ethephon (ET)	**25.7**	4.27862E−11	**15.5**	0.000	**4.4**	0.013581983	2.2	0.115	**8.0**	0.000	1.4	0.248
Clone × Tapping	**14.5**	6.18611E−11	**27.4**	0.000	0.8	0.548009233	0.9	0.492	**4.5**	0.001	0.3	0.860
Clone × ET	**4.6**	0.00124788	1.5	0.196	0.4	0.810645755	0.8	0.533	0.5	0.742	0.2	0.950
Tapping × ET	1.3	0.289389056	0.6	0.672	0.5	0.740940139	1.6	0.169	1.1	0.361	0.2	0.961
Clone × Tapping × ET	6.8	2.79086E−08	4.2	0.000	0.3	0.957317842	0.2	0.989	1.1	0.381	0.1	1.000

Tukey test on expected marginal mean values computed by ANOVA for clone x tapping × ethephon interaction show that clone PB 260 has a significantly higher DCL values than the other clones for all treatments except for d2 and d4 without stimulation (Supplementary Table 4, column G). It also has a lower sucrose content compared to other clones. For TSC, Pi and RSH, the differences between clones are not significant. Clone RRIM 600 stands out for its low DCL and high sucrose content for all treatments (values from 4.4 to 7.8 mM), as well as high yield and Pi for d1.

Clone SP 217 is an intermediate clone. For this clone, higher DCL values (37.1 and 44.1%) appear for d1 with stimulation by ethephon 12 and 24 times a year. These intermediate DCL values are due to a high DCL occurring after 2 years of tapping (Supplementary Table 3). For other parameters (TSC, sucrose, Pi and RSH), their values are in between the two other clones.

This PT1 experiment showed that high DCL can be induced rapidly in the TPD susceptible clone PB 260 when using a high-tapping frequency harvesting system with ethephon stimulation. The clonal effect is highly significant for DCL. High tapping frequency and ethephon stimulation were applied on the PT2 trial in order to induce high DCL in susceptible clones and validate the previous results.

**Figure 1:**
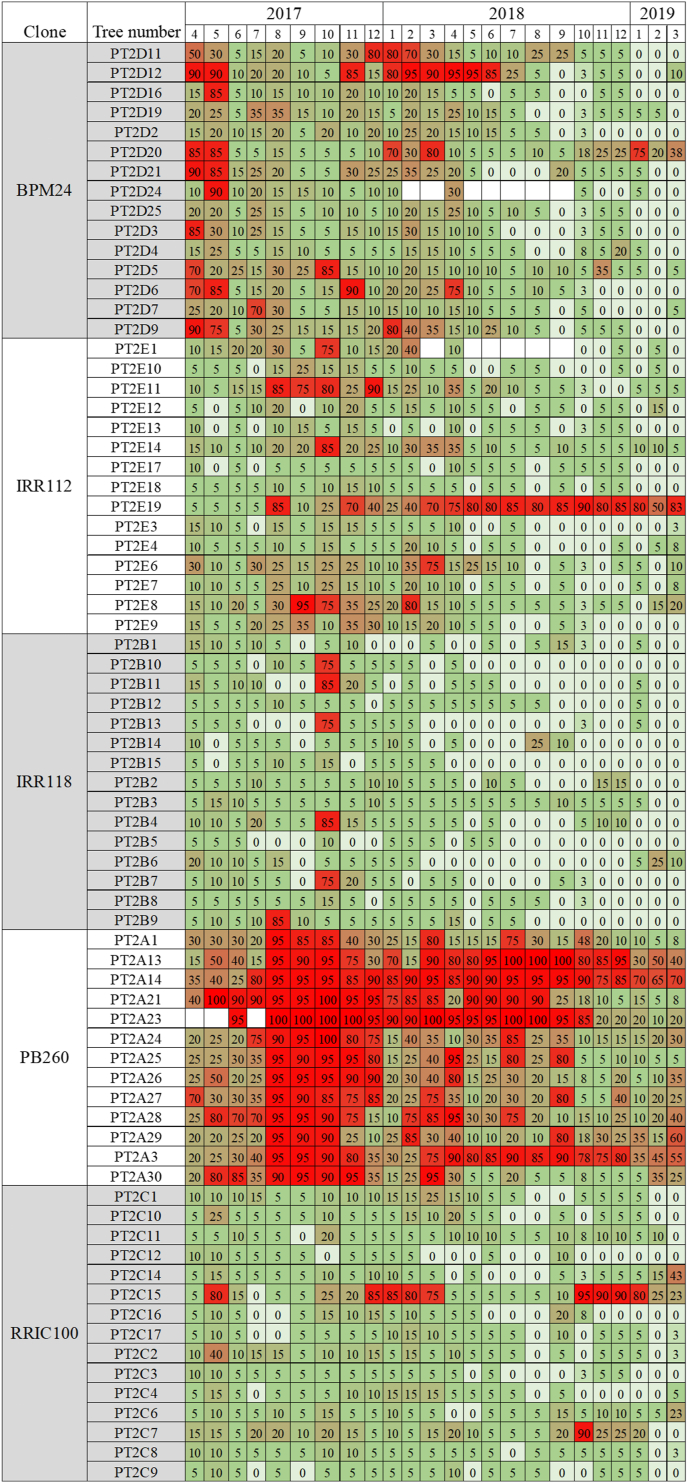
Percentage of dry cut length (DCL) determined every month for 24 months for each tree of the PT2 trial consisting of 5 rubber clones (BPM 24, IRR 112, IRR 118, PB 260, RRIC 100). Gradient of DCL was noted from 0% DCL in white to >60% DCL in red.

**Figure 2:**
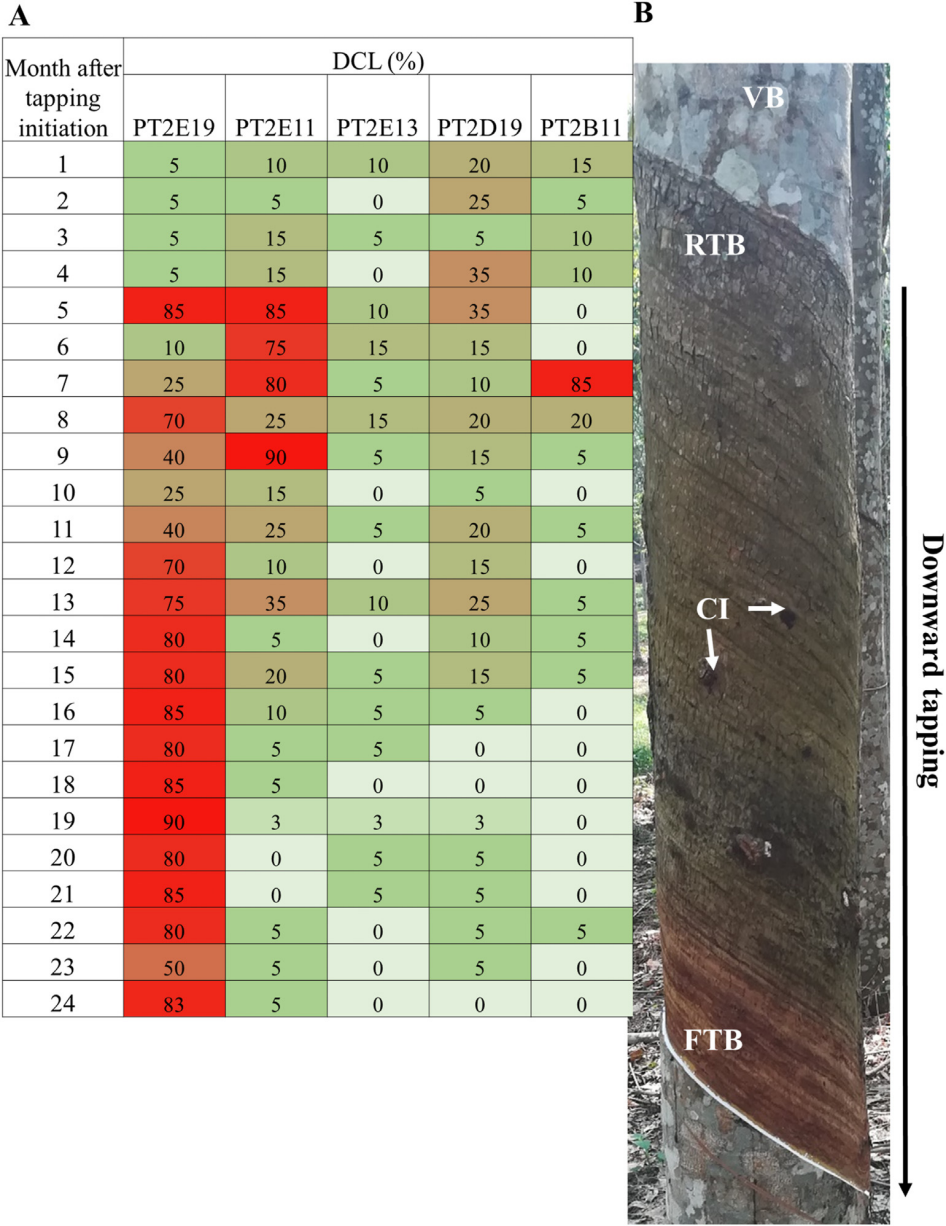
(A) Main DCL profiles observed during 24 months after tapping initiation for the three DCL classes. (1) low DCL (mean < 20%) as tree PT2E13; (2) low to medium DCL (<50%) as tree PT2D19; (3) low to high DCL (up to 100%) as tree PT2E19; (4) transient high DCL as tree PT2E11. Trees were tapped every day from Monday to Saturday and ethephon 2.5% was applied to the tapping cut once a month (d1 ET 2.5 % 12/y). (B) Tapped panel after 24 months of downward tapping using harvesting system d1 ET 2.5 % 12/y. VB: virgin bark; RTB: regenerated tapped bark; FTB: freshly tapped bark. CI: trace of slight vascular cambium injury.

DCL was monthly monitored for each tree during two years in the PT2 trial (Figure 1, Supplementary Table 5). As for PT1, all trees have at least a dry area on the tapping cut at a given period of time during the two-year monitoring. Each tree was labelled by the k-means clustering method as high DCL (class 1), medium DCL (class 2), low DCL (class 3). The mean of DCL ranges from 38-80% for class 1, 16–35% for class 2, and 2–16% for class 3. Several profiles of DCL expression can be observed (Figure 2A). For trees of class 1, several successive high DCL percentage can be observed such as for tree PT2E19. For class 2, trees can have a transient period of time of one to three months with high DCL (see example of tree PT2E11). For class 3, trees can have a low DCL between 0 to 15% as PT2E13, or a low DCL with a transient medium DCL (20–40%) for several months as PT2D19, or a low DCL with a peak for one month at a high level (70–90 %) as PT2E1. Bark consumption after 2 years can reach 92 ± 3 cm for a high tapping frequency in d1 (Figure 2B, Supplementary Table 6). Bark regeneration is initiated rapidly after the tapping. Fully regenerated tapped bark (RTB) can be seen on top of the panel while freshly tapped bark (FTB) is not yet regenerated.

**Figure 3:**
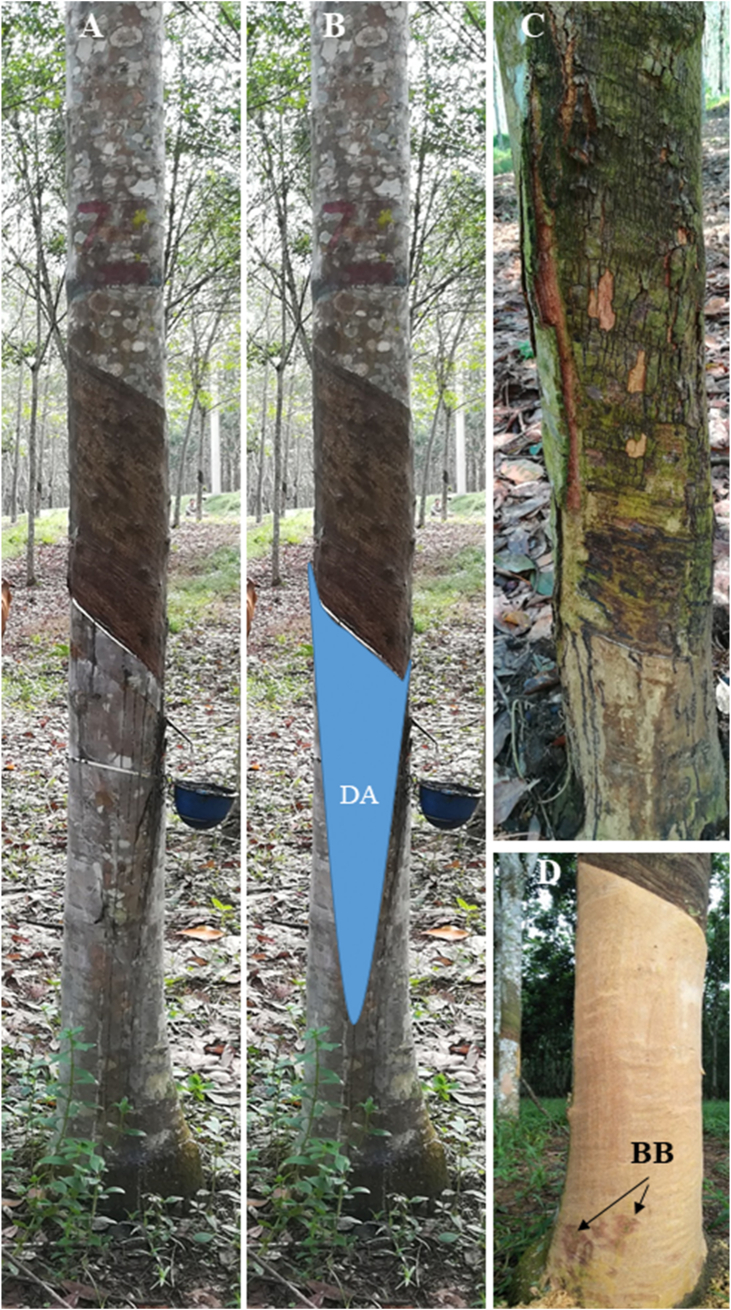
(A) Tapped trees panel after 18 months of downward tapping using intensive harvesting system d1 ET 2.5 % 12/y in trial PT2. (B) In blue, putative drainage area (DA) from where the latex flows. (C) Example of tapped panel showing bark cracks for some high DCL trees. (D) Identification of brown bast spots observed after bark scraping of the panel below the tapped area in some high DCL trees.

Latex collected after tapping is supposed to come from a drainage area consisting of the articulated and anastomosed laticifer network (Figure 3A and B). At the end of the 2-year monitoring, some trees of clone PB 260 showed advanced brown bast symptoms with bark cracking and degeneration (Figure 3C). Most of brown bast spots can be observed only after bark scraping of the virgin bark below the tapped panel (Figure 3D). Brown bast intensity was divided into three classes based on the number of brown bast spots using K-means clustering (Supplementary Table 5). Of the 73 analysed trees, 14 trees have brown bast spots.

**Figure 4:**
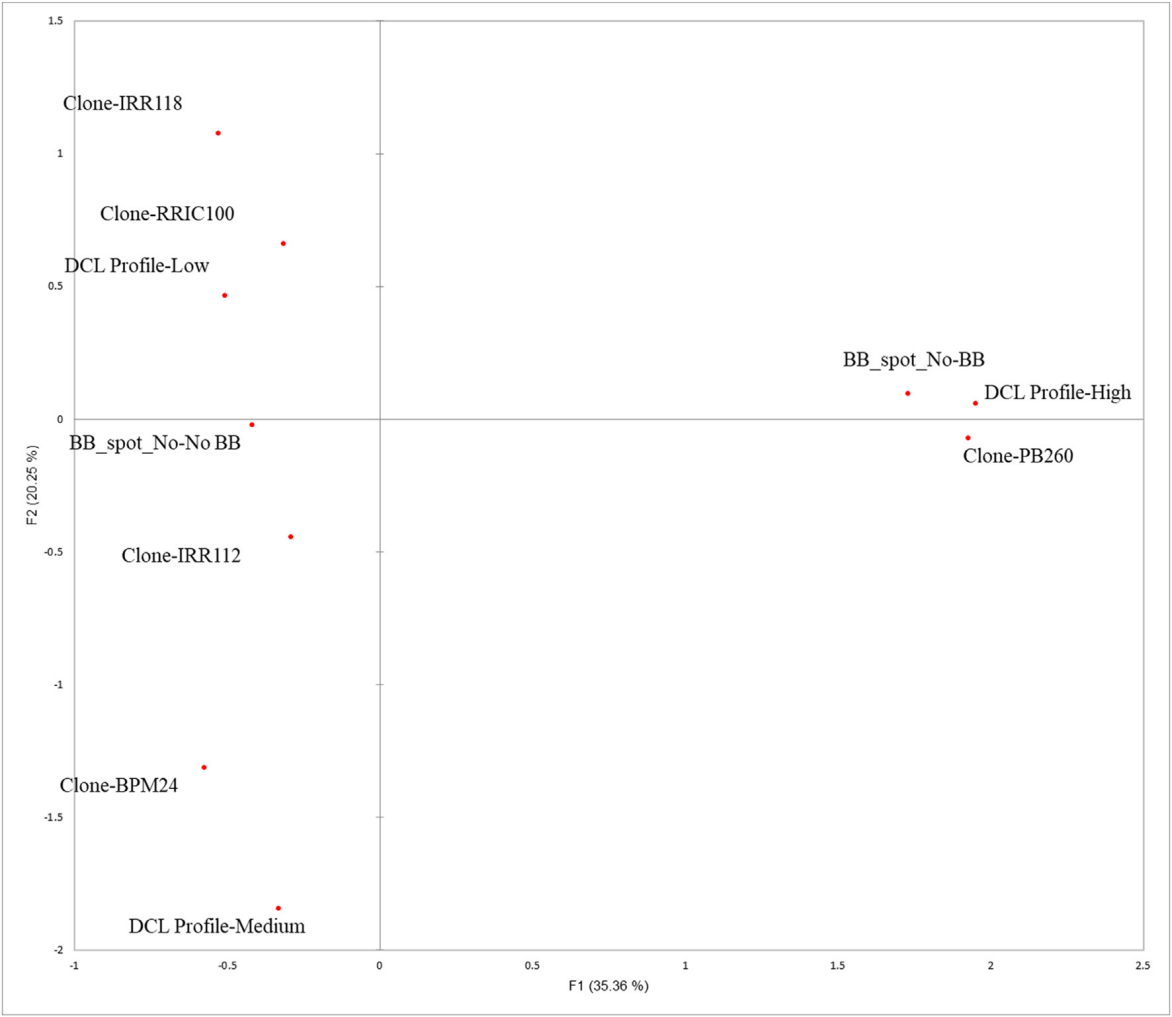
Multiple Correspondence Analysis (MCA) based the qualitative data of Supplementary Data 5.

The number of brown spots is linked to DCL level: eleven brown bast spots were seen for trees with high DCL, 1 spot with medium DCL, 2 with low DCL. To study more accurately the links between clone, DCL and BB classes, multiple correspondence analysis (MCA) was performed (Figure 4). On axis F1 (35% of total variance), occurrence of BB spots appears associated with both high DCL and clone PB 260. On the opposite side, absence of BB is associated with low and medium DCL as well as other clones. On axis F2 (20% of total variance), low DCL is associated with clones IRR 118 and RRIC 100, which are opposed to clone BPM 24 and medium DCL.

**Figure 5:**
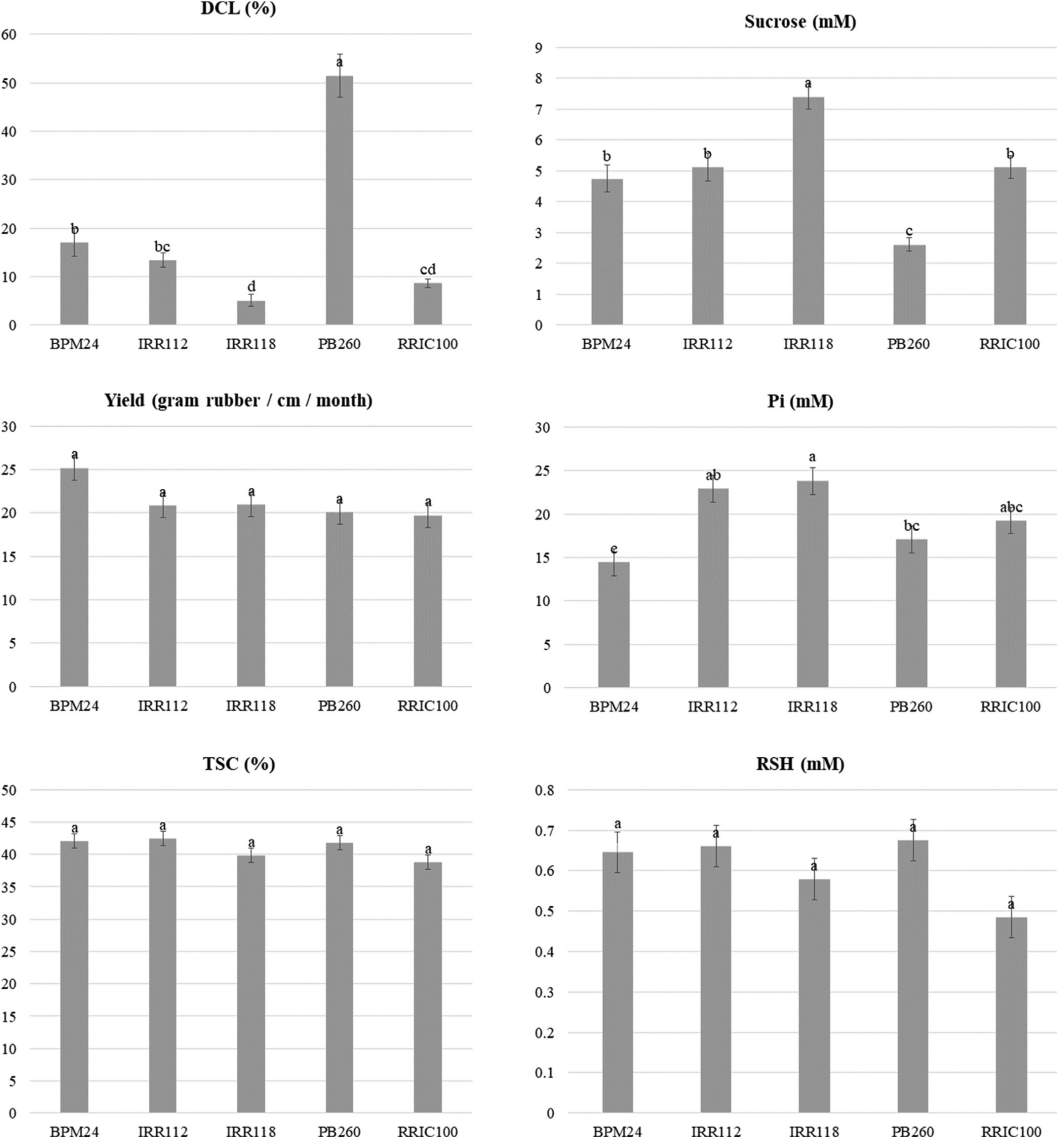
Mean and standard error for yield, dry cut length (DCL), total solid content (TSC), sucrose, inorganic phosphorus (Pi), and reduced thiols (RSH). Means of 15 trees for each of the 24 months were used for the calculation of the means combining measurements of all dates for each clone. ANOVA was performed with the Tukey test (95%). The variability of DCL and sucrose being unequal across the range of values, heteroscedasticity option was selected with method HC1.

**Figure 6:**
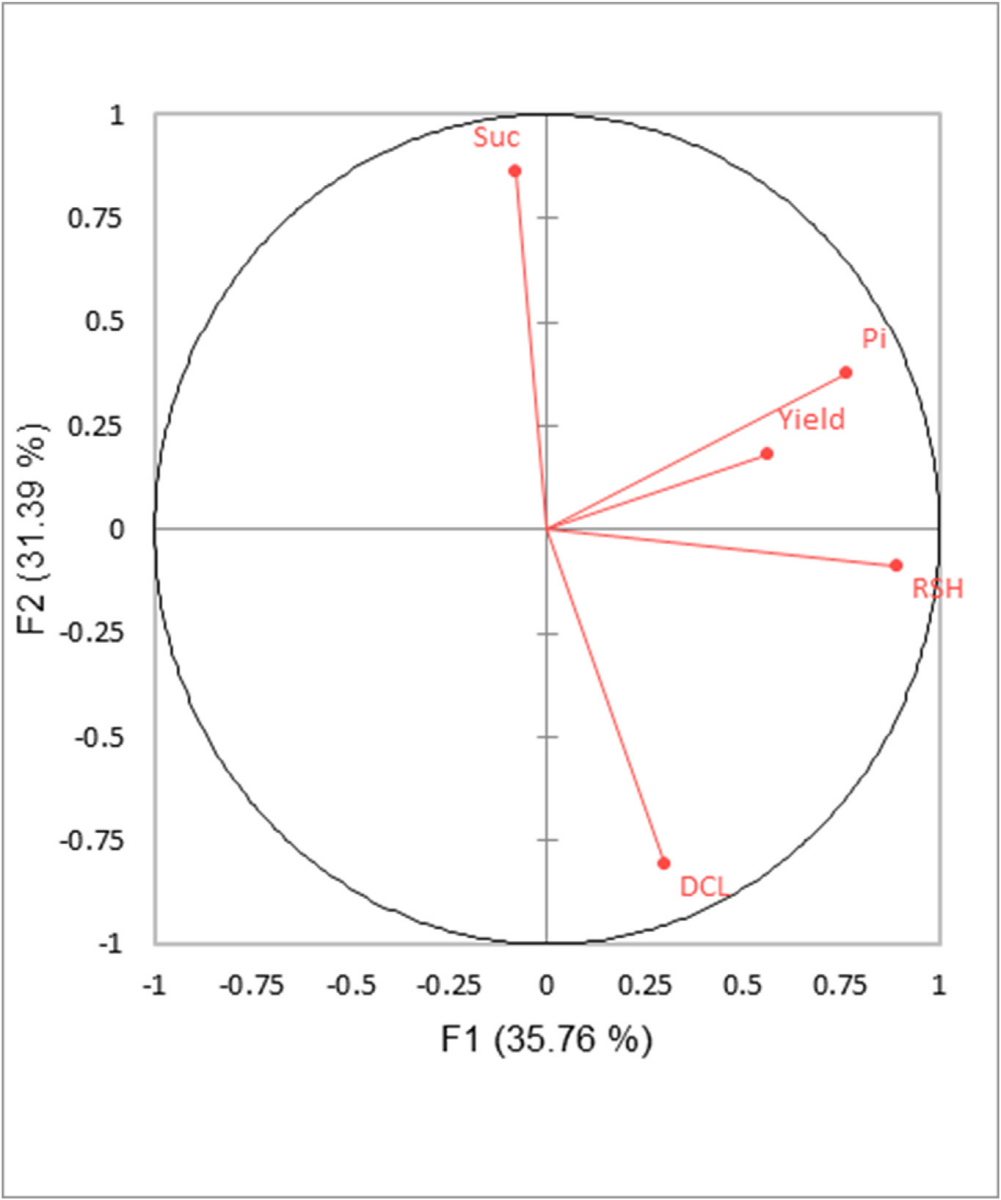
Principal Component Analysis (PCA) of mean and residues data for yield, dry cut length (DCL), sucrose, inorganic phosphorus (Pi), and reduced thiols (RSH).

**Figure 7:**
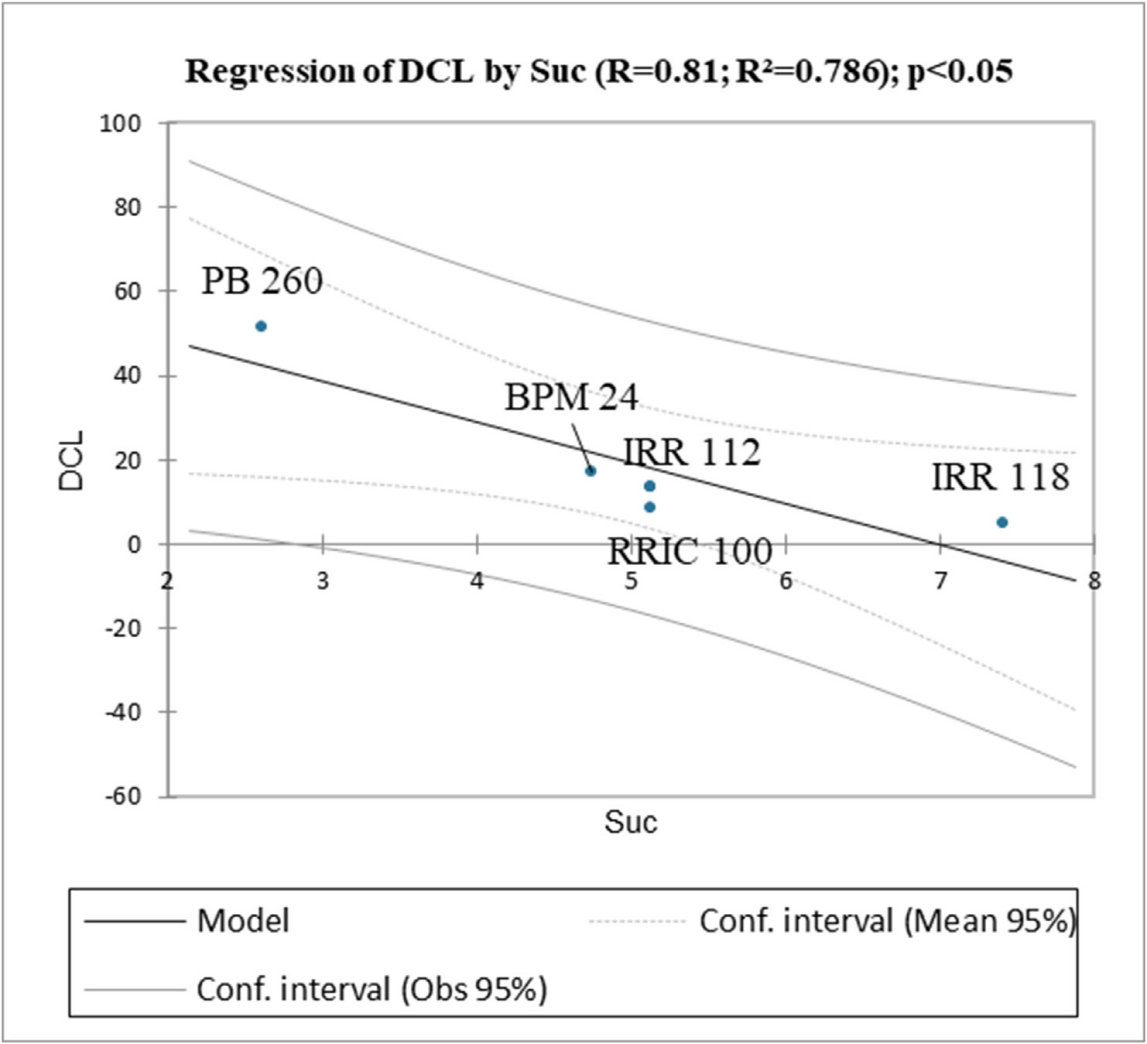
Linear regression of dry cut length (DCL) by sucrose content for the mean value for clones.

Latex yield and LD were monthly monitored for 15 trees per clone of the PT2 trial during the 2-year period (Figure 5). Clone PB 260 had the highest level of DCL (55%) compared to other clones which range from 5 to 17%. By contrast, clone PB 260 had the lowest content in sucrose (2.6 mM). Clone IRR 118 had both the highest Pi (23.8 mM) and sucrose (7.4 mM) contents compared to other clones. No significant difference between clones (P > 0.05) was observed for yield, TSC and RSH. A PCA was performed in order to visualize the association between these variables (Figure 6). TSC data were discarded from the PCA analysis because of the large number of missing data, especially in 2017. PCA exhibits two independent components with eigen values higher than one (1.78 and 1.57, respectively). The first component expresses the positive correlation between Pi and RSH (coordinates 0.77 and 0.89 respectively). On the 2^nd^ component, sucrose (0.86) is negatively correlated to DCL (−0.81). The linear regression of the mean values of monthly sucrose data on DCL is significant with a high correlation between these variables (R = 0.81) (Figure 7). The regression of Pi on RSH is not significant.

## 4 Discussion

Tapping Panel Dryness is a physiological syndrome affecting latex production. Both preventing TPD in plantations and breeding for TPD-tolerant clones are still challenging, especially in a context of climate change. In this study, the dynamic analysis of TPD reveals new insights on the establishment of TPD, the relation between latex metabolism and the tolerance of clones to TPD as well as the ability to perform an early assessment of clones for TPD susceptibility.

### 4.1 Dynamic analysis of DCL, latex yield and LD reveals a complex establishment of TPD

In rubber plantations, TPD-affected trees are identified by tappers only when the latex yield is very low in the cup. This loss of production is associated with a late phase of TPD occurrence consisting of high DCL and brown bast occurrence. Agronomy literature often mentions the dry tree incidence corresponding to the number of dry trees per hectare.

Physiological and molecular studies have also a binary approach considering healthy and dry rubber trees. Based on the determination of DCL, some authors have described TPD ranging from healthy, slight or mild, intermediate or moderate, and severe occurrence (Guo et al., 2016; Liu et al., 2018; Putranto et al., 2015) or by class from 1 to 6 (Rukkhun et al., 2020). Apart from Putranto and collaborators, the other authors just classed TPD trees before sampling or recorded DCL twice a year.

In this study, monthly observation of DCL on the 147 trees of the PT1 (74 trees) and PT2 (73 trees) trials shows that even the trees considered to be free of TPD have small and localized dry spots. Small dry spots may appear on the tapped panel regardless of clone, harvesting system and season. Conversely, high DCL occurrence is significantly associated with clone and harvesting system factors (Table 1). During the first years after tapping, 3 years for PT1 and 2 years for PT2, latex yield did not significantly dropped, even in trees with high DCL. This highlights that latex comes from a large drainage area (Figure 3B). Latex flows from areas without or with mild TPD symptoms masking the effect of TPD-affected areas in terms of latex volume.

Dry spots can appear in all trees during the rubber production cycle. Monitoring for DCL can help assess the level of its occurrence and determine if there is a risk of TPD affecting the latex production.

### 4.2 Sucrose content is a physiological marker associated with clonal tolerance to TPD

High latex metabolism rubber clones are often considered as TPD-susceptible clones (Tistama et al., 2019). The level of latex metabolism is usually reflected by the inorganic phosphorus content. In the PT1 trial, the high latex metabolism clone PB 260 had TPD trees for different harvesting conditions while the low latex metabolism clone SP 217 showed no TPD incidence in the standard harvesting system (Putranto et al., 2015). Two rubber clones selected by the Indonesian Rubber Research Institute, IRR 112 and IRR 118, have a high latex metabolism. Unlike clone PB 260, these clones had a very low TPD incidence during the two-year monitoring of the PT2 trial. This result shows that some high latex metabolism can better stand intensive harvesting system without or with low TPD occurrence.

Interestingly, clone IRR 112 and especially clone IRR 118 had high sucrose contents, 5.1 mM and 7.4 mM, respectively, compared to other clones. The clonal sucrose concentration in latex was inversely correlated to the DCL level (Figure 7). This robust clonal value was calculated from the values of 15 trees during two years monitoring in order to avoid any specific and temporary physiological effects. Sucrose is a source of energy and carbon in laticifers. Sucrose plays an essential role in rubber biosynthesis and defence mechanism. Recurrent stress from latex regeneration and flow as well as mechanical wounding by tapping require high level of protecting agents to prevent oxidation and consequent TPD occurrence. RSH content is usually considered as an indicator of membrane protection from ROS in laticifers (Zhang et al., 2017). In this study, RSH content was not significantly associated with TPD although RSH content trends to drop in the second year after intensive harvesting system d1 ET 12/y. A literature survey reveals the absence of consistent effect of RSH content with regard to stimulation, fertilization (Chotiphan et al., 2019) and a strong seasonal effect (Rukkhun et al., 2020). Many studies reported that some physiological parameters can be associated with TPD such as proline or latex copper (Yusof et al., 1995), proteins (Kun et al., 2014), inorganic phosphorus, Mg^2+^, lutoid bursting index (Guo et al., 2016). However, the dissemination and application of these results remain limited. The high variability of physiological parameters with regard to seasonal effects, and the buffering effect due to the large drainage area on the tapped panel can make difficult the repeatability of these findings. In the case of daily tapping such as d1 ET 12/y, the latex is not fully regenerated and can lead to unstable latex parameters. LD analysis is recommended during the peak season of latex production, which combines a full canopy and good level of rainfall. To date, sucrose content is widely used as LD parameters and its values are well interpreted in terms of yield potential. In this study, the sucrose was negatively associated with TPD despite the above cited constraints (Figure 7).

### 4.3 High tapping frequency with ethephon stimulation for phenotyping rubber populations

The effect of intensive tapping has long been known on the occurrence of TPD (Eschbach et al., 1989). In daily tapped trees, latex regeneration cannot be completed and causes stress in laticifers. Conversely, TPD can probably be reduced by low tapping frequency (Traoré et al., 2011). TPD incidence is often observed in smallholdings, which use high tapping frequency (Rukkhun et al., 2020). In Thailand, the heavy rainfall and the increase in rainfall events lead farmers to use high tapping frequency systems to compensate for the reduction of tapping days due to the rainfall (Chantuma et al., 2011). This practice tends to induce high levels of TPD, increase bark consumption and shorten the life cycle of rubber plantations (Obouayeba et al., 2009; Rukkhun et al., 2020).

In this paper, the application of high tapping frequency and ethephon stimulation (d1 ET 12/y) is a way to accelerate the onset of TPD and facilitate its study. Most of the trees from clone PB 260 had an early high DCL (before 6 months after opening) in the two independent experiments PT1 and PT2 conducted at 6 years apart. According to Putranto and collaborators (Putranto et al., 2015), high tapping frequency without stimulation also induces high DCL. By contrast a higher frequency of stimulation (24 times a year) did not accelerate the occurrence. The use of stimulant 12 times a year is also simpler way to synchronize the trial with the recommendation for latex diagnosis (3 weeks without stimulation). This finding suggests that such an intensive harvesting system may be used to phenotype *Hevea* populations and identify TPD susceptible genotypes.

Although agronomists have observed a clonal susceptibility to TPD, its heritability has never really been estimated by geneticists. The first reason is the low natural occurrence of TPD in plantations for standard recommended harvesting systems (about 1% per year). However, a certain level of clonal variation of TPD rate was determined in large-scale clone trials for six rubber clones (Elabo et al., 2019). The second reason is related to the high environmental influence. Omokhafe and collaborators suggested that conducting a phenotyping of a large number of clones could enhance genetic variability, heritability and genetic progress for low incidence of tree dryness (Omokhafe, 2004; Omokhafe and Aniamaka, 2000). Some authors reported the identification of random amplified polymorphic DNA markers linked to tolerance to TPD (Li et al., 2012; Thulaseedharan et al., 2002). To our knowledge, no application has been published to date.

## 5 Conclusion

This paper set out a new perception of Tapping Panel Dryness. All trees develop more or less important dry spots without systematically affecting latex production whatever the harvesting system and the season. The level of DCL changes over time. The irreversible form of TPD (brown bast) sets in when high DCL occurs. The application of an intensive harvesting system (d1 ET 12/y) induces early TPD occurrence and homogenizes the clonal response in the TPD susceptible clone PB 260. These results suggest that this methodology be applied to phenotype *Hevea* populations so as to identify TPD-susceptible genotypes. The genetic × environment interaction may also be analysed in order to make this study more robust. Sucrose content is an essential latex diagnosis parameter for estimating yield potential and responsiveness to ethephon stimulation. Sucrose in latex was here negatively associated with TPD susceptibility. In a context of climate change, breeding for TPD-tolerant clones is becoming a matter of emergency. Studies on molecular and genetic bases of TPD should be facilitated by using intensive harvesting system and such a physiological marker. These findings should lead to applications especially in breeding programmes and genetics studies.

## Declaration

### Author contribution statement

Pascal Montoro, Fetrina Oktavia: Conceived and designed the experiments.

Eva Herlinawati, Sigit Ismawanto, Afdholiatus Syafaah, Martini Aji: Performed the experiments.

Pascal Montoro, Eva Herlinawati, Eric Gohet, Michel Giner, Albert Flori: Analyzed and interpreted the data.

Eva Herlinawati, Afdholiatus Syafaah, Martini Aji: Contributed reagents, materials, analysis tools or data.

Pascal Montoro: Wrote the paper.

### Funding statement

Dr. Fetrina Oktavia was supported by Indonesian Rubber Research Institute.

Mr. Pascal Montoro was supported by 10.13039/501100007204CIRAD.

### Data availability statement

Data included in article/supplementary material/referenced in article.

### Declaration of interest’s statement

The authors declare no conflict of interest.

### Additional information

No additional information is available for this paper.
